# Supplementary material for: Enhancing unity-based AR with optimal lossless compression for digital twin assets
Source: PLoS One. 2024 Dec 19;19(12):e0314691. doi: 10.1371/journal.pone.0314691 (PMC11658801; doi:10.1371/journal.pone.0314691)
Supplement: Attachment — Submitted filename: Rebuttal Letter_PLOS ONE.pdf [file pone.0314691.s001.pdf]

Rebuttal Letter

Subject: Rebuttal of Reviewer Comments on Manuscript [PONE-D-23-39558] - [EMID:5b75880c1a491e5a]

Dear Editor/Reviewer's,

I hope this response finds you well. We appreciate the constructive feedback provided by the reviewers and have carefully addressed each of the comments. Here is our response to the specific suggestions:

1. **Missing Figures in the Text:**

Reviewer Comment: All figures mentioned in the text are missing.

Authors' Comment: We have successfully addressed this issue by embedding all images in the manuscript after a thorough check. The images have been converted to meet PLOS ONE requirements using the Preflight Analysis and Conversion Engine (PACE) digital diagnostic tool.

2. **Inconsistency in Displayed Error Metrics:**

Reviewer Comment: Although the Residual Standard Error (RSE) is used and referred to in the text, in tables 6, 7, 8, and 9, the Root Mean Square Error (RMSE) is displayed instead. Please change accordingly.

Authors' Comment: We have carefully corrected and updated all relevant values in tables and images. The Residual Standard Error (RSE) has been consistently used as per the reviewers' suggestion, replacing the Root Mean Square Error (RMSE) in tables 6, 7, 8, and 9.

3. **Inclusion of Charts to Complement Tables:**

Reviewer Comment: Please consider including charts to add to tables 7, 8, and 9 to clarify differences to the reader and accomplishments regarding, for example, RSE per compression techniques.

Authors' Comment: New bar charts have been created, embedded, and referred to in the context of tables 7, 8, and 9. These charts aim to provide a clearer visualization of differences in RSE values for various compression techniques. Additionally, the new plots have been designed to display the comparison of different methods in the same graph, with equations and additional statistical terms included for a more comprehensive understanding.

We believe that these revisions not only address the specific concerns raised by the reviewers but also enhance the overall clarity and quality of the manuscript. We sincerely thank the reviewers for their valuable input and the opportunity to improve our work.

Thank you for your consideration.

Sincerely,

Mohammed Hlayel
