# Supplementary material for: Enhancing unity-based AR with optimal lossless compression for digital twin assets
Source: PLoS One. 2024 Dec 19;19(12):e0314691. doi: 10.1371/journal.pone.0314691 (PMC11658801; doi:10.1371/journal.pone.0314691)
Supplement: Attachment — Submitted filename: Response to Reviewer_1.pdf [file pone.0314691.s002.pdf]

Response to Reviewer #1

Thank you for your valuable feedback on our paper. We have addressed each of your points in detail below, providing additional clarifications and extending our discussion to improve the manuscript's depth and clarity.

Methodology for Evaluating Compression Methods

Our evaluation of compression methods involved a comprehensive and multifaceted approach, focusing on key performance indicators (KPIs) relevant to AR-based mobile applications. Here are the detailed steps and considerations in our methodology:

1. **Selection Criteria for Compression Algorithms**: We chose a variety of widely-used lossless compression algorithms based on their popularity, differing compression strategies, and suitability for different asset types common in AR applications. The algorithms tested include:

    - **LZ4**: Known for its extremely fast compression and decompression speeds.
    - **LZMA**: Utilized in 7-Zip for its high compression ratios, albeit with slower decompression.
    - **Zip and GZip**: Classic algorithms with balanced performance.
    - **Fast LZ**: Optimized for speed, making it suitable for real-time applications.
    - **Brotli**: Developed by Google, offering a good balance between speed and compression efficiency.
    - **7-Zip**: An LZMA-based algorithm known for its high compression efficiency.

2. **Experimental Setup**:

    - **Benchmarking Framework**: We developed a robust benchmarking framework within the Unity environment, integrating it with the Vuforia AR Engine to simulate realistic usage scenarios. This framework enabled us to apply compression to various assets such as textures, 3D models, audio files, and multimedia content.
    - **Test Assets**: A curated set of standardized assets representative of typical AR applications was used. This

included high-resolution textures, complex 3D models, and interactive multimedia files. Each asset type posed different challenges for compression algorithms, providing a thorough evaluation.

3. **Performance Metrics and Data Collection**:

    - **Compression Ratio**: Defined as the ratio of the compressed size to the original size of the asset. This metric directly influences the storage efficiency and the amount of data transferred.
    - **Decompression Time**: The time required to restore compressed assets to their original form. We measured this using high-resolution timers within the Unity environment to capture accurate decompression timings.
    - **Memory Usage**: We monitored the memory footprint during both compression and decompression processes. This included peak memory usage and average memory consumption, providing insights into the resource efficiency of each algorithm.
    - **CPU Load**: CPU utilization was measured using profiling tools available in Unity, focusing on both peak and average load during decompression. This helped assess the computational overhead and its potential impact on device performance.

4. **Testing Scenarios**:

    - **Simulated Usage**: We simulated real-world usage scenarios by deploying compressed assets in interactive AR environments. This included scenarios where assets are frequently loaded and unloaded to mimic typical user interactions.
    - **Device Variability**: To ensure the results were generalizable, we tested the algorithms on a range of mobile devices with varying hardware capabilities. This included high-end smartphones, mid-range devices, and low-power tablets, covering different processor architectures and memory configurations.

5. **Data Analysis**:

- **Statistical Analysis**: We conducted a statistical analysis to evaluate the performance of each compression algorithm across different metrics. This included calculating mean, median, and standard deviation for decompression times, memory usage, and CPU load.
- **Comparative Evaluation**: A comparative evaluation was performed to identify the strengths and weaknesses of each algorithm. We used visualizations such as bar charts and scatter plots to illustrate performance trade-offs.

Deeper Insights into the Results and Practical Implications

1. **Compression Ratio**:

   - **Brotli and LZMA**: These algorithms achieved the highest compression ratios, significantly reducing file sizes by up to 40-60%. However, their higher complexity led to slower decompression speeds.
   - **LZ4 and Fast LZ**: Although offering lower compression ratios (around 20-30%), these algorithms excelled in decompression speed, making them ideal for real-time applications where quick access to assets is critical.

2. **Decompression Time**:

   - **LZ4**: Provided the fastest decompression times, often under a second for medium-sized assets. This made it particularly suitable for applications requiring rapid content loading, such as interactive games or live AR experiences.
   - **LZMA**: While slower, LZMA's decompression times were acceptable for scenarios where the frequency of asset access is lower, such as initial application loading or background data synchronization.

3. **Memory Usage**:

   - **Brotli and LZMA**: These algorithms required more memory during decompression due to their sophisticated compression techniques. Their memory footprint was manageable but higher than faster algorithms.

- **LZ4 and Fast LZ**: Demonstrated lower memory usage, aligning well with the constraints of mobile devices, particularly those with limited RAM.

4. **CPU Load**:

- **LZ4 and Fast LZ**: Exhibited minimal CPU load, making them suitable for devices with lower processing power. This is crucial for maintaining overall application responsiveness.
- **Brotli and LZMA**: Induced higher CPU loads due to their intensive decompression processes, which might impact performance on less powerful devices.

Practical Implications

1. **Balancing Trade-Offs**:

- **Interactive Applications**: For AR applications that require frequent and rapid asset loading, developers should prioritize algorithms like LZ4 or Fast LZ, which offer quick decompression times and low CPU usage.
- **Storage-Constrained Scenarios**: In situations where storage space is a premium, such as on devices with limited internal storage or applications that download large amounts of data, Brotli or LZMA might be more appropriate despite their slower decompression times.

2. **Device Considerations**:

- **High-End Devices**: On devices with ample processing power and memory, more complex algorithms like Brotli and LZMA can be employed without significantly impacting performance.
- **Low-End Devices**: For devices with limited resources, algorithms with low memory and CPU requirements, such as LZ4 and Fast LZ, should be preferred to maintain a smooth user experience.

3. **User Experience**:

- **Fast Decompression**: Users expect quick and seamless interactions in AR applications. Thus, algorithms that offer fast decompression times directly contribute to a better user

experience by reducing wait times and improving the responsiveness of the application.

4. **Development Workflow**:

    - **Automated Testing**: Implement automated testing frameworks to evaluate the performance of different compression methods regularly. This ensures that the chosen compression strategies remain optimal as the application evolves.
    - **Profiling and Optimization**: Utilize profiling tools to continuously monitor and optimize memory usage and CPU load during development, ensuring that the compression methods do not introduce unexpected performance bottlenecks.

Actionable Recommendations for Developers

1. **Algorithm Selection**: Based on the application's needs, select compression algorithms that offer the best trade-off between compression efficiency and decompression speed. LZ4 or Fast LZ for speed-critical applications, Brotli or LZMA for storage optimization.
2. **Dynamic Adaptation**: Consider implementing a dynamic compression strategy where different algorithms are applied based on the type of asset and its usage frequency. Static assets can be compressed with higher-ratio algorithms, while dynamic or frequently accessed assets use faster algorithms.
3. **Hybrid Compression**: Explore hybrid compression techniques that combine the strengths of multiple algorithms. For instance, using a fast algorithm for initial decompression followed by a more efficient algorithm for long-term storage.
4. **Real-Time Profiling**: Integrate real-time profiling tools within the development pipeline to continuously monitor the performance impact of compression algorithms, allowing for adjustments based on real-world usage patterns.
5. **Future-Proofing**: Stay updated with advancements in compression technology and regularly evaluate new algorithms that may offer better performance or efficiency improvements.

Accuracy and Reliability of Statistical Modelling

Our statistical models aimed to predict the impact of compression methods on the performance of AR-based applications. These models focused on:

- **RAM and CPU Requirements**: Estimating the computational resources required for decompression based on asset type and size.
- **Bundle Size Estimation**: Predicting the size of compressed bundles relative to the content, aiding in planning and resource allocation during development.

The tables and graphs were reviewed and compared precisely. To enhance the accuracy and reliability of these models:

1. **Comprehensive Datasets**: We utilized a diverse set of assets representing different data types and sizes to ensure the models are robust and applicable across various scenarios.
2. **Cross-Validation**: Applied cross-validation techniques to assess model performance and mitigate overfitting. This involved partitioning the data into training and testing sets and validating the models against unseen data.
3. **Real-World Validation**: The models were tested against real-world application scenarios to verify their accuracy in predicting performance impacts, ensuring they reflect practical conditions.

Limitations and Future Research Directions

1. **Scope of Compression Algorithms**: While our study focused on several popular algorithms, future research could extend to other compression techniques such as:

    - **Zstd**: Known for its speed and good compression ratios.
    - **Snappy**: Optimized for very high-speed compression and decompression.
    - **LZHAM**: A high-ratio alternative to LZMA with faster decompression.

2. **Cloud-Based Testing**: Future research could explore cloud-based testing environments rather than local FTP. This approach would simulate real-world scenarios where assets are stored and accessed via cloud services, providing insights into how compression

algorithms perform under network latency and bandwidth constraints.

3. **Live Decompression**: Investigate the feasibility of live decompression techniques where assets are decompressed on-the-fly during streaming. This could be particularly beneficial for applications with dynamic content or live updates.
4. **Real-Time Adaptation**: Explore real-time adaptive compression techniques that dynamically select the most appropriate compression method based on current device state, network conditions, and user interaction patterns.
5. **Advanced Compression Techniques**: Examine advanced compression methods such as neural compression or context-based algorithms that could offer better performance for specific data types.
6. **Integration with Emerging Technologies**: With the advent of technologies like 5G and edge computing, integrating these compression strategies with cloud-based systems could further enhance performance by offloading decompression tasks to more powerful edge servers.

We have made these enhancements and clarifications to address your feedback comprehensively. We believe these changes significantly improve the clarity and depth of our paper. Thank you for your constructive comments, and we look forward to any further suggestions you may have.
