# Supplementary material for: Enhancing unity-based AR with optimal lossless compression for digital twin assets
Source: PLoS One. 2024 Dec 19;19(12):e0314691. doi: 10.1371/journal.pone.0314691 (PMC11658801; doi:10.1371/journal.pone.0314691)
Supplement: Attachment — Submitted filename: Response to Editor.pdf [file pone.0314691.s003.pdf]

Response to Editor's Comments

We sincerely appreciate the thorough feedback provided by the reviewer. Below is our point-by-point response to the comments, addressing each concern in the revised manuscript.

---

1. The Authors not properly follow the PLOS ONE Paper format.

The manuscript has been revised to fully adhere to the PLOS ONE submission guidelines as outlined in PLOS ONE's Manuscript Organization section. We ensured that the paper was organized according to the required structure, with sections properly aligned with PLOS ONE standards, including title, abstract, introduction, methods, results, discussion, and conclusion.

---

2. The whole paper flow control and connectivity is ambiguous; make it readable.

We acknowledge that the nature of the research involves multiple methodologies, technical parameters, and the application of various statistical models, which can make the presentation complex. However, we have significantly revised the manuscript's structure to ensure clarity and coherence. Despite the technical intricacies, we enhanced the logical transitions between sections, allowing readers to follow the paper's flow more easily. We provided a smoother, more organized narrative by carefully structuring the introduction, literature review, methodology, and results sections. This revised flow facilitates a step-by-step understanding of the research while maintaining the depth and technical rigor required for the study.

---

3. Make sure the paper format in terms of Introduction, Literature, Proposed Methodology, and Results and Discussion, etc.

We have revised the manuscript to ensure compliance with standard academic structure, organizing the paper into the following sections:

- **Introduction**: Provides the background and context.
- **Literature Review**: Discusses related research.
- **Materials and Methods**: Details of the experimental setup and methods.
- **Results and Discussion**: Presents and analyzes the results.
- **Conclusion and Future Direction**: Summarizes findings and suggests future research directions.

This structure is in alignment with PLOS ONE's guidelines for manuscript organization.

---

4. Conduct some critical analysis to prove the strength of the proposed scheme.

A comprehensive critical analysis has been incorporated into each paper discussion section. For instance, in the Compression Ratio section, we critically examined the efficiency of various compression algorithms, assessing their performance relative to each other based on different dataset sizes. The Total Time section provided insights into the real-time applicability of our method, comparing the time efficiency across different algorithms. In the Decompression Time section, we critically evaluated the proposed method's decompression speed and CPU performance, identifying where our approach offers measurable improvements over existing methods. Similarly, the RAM Consumption analysis confirmed the scheme's robustness by demonstrating its ability to maintain stable performance across varying bundle sizes and data types. These critical insights were supported by statistical evidence and comparative data, proving the effectiveness of our approach.

We have included a comprehensive critical analysis in the results and discussion sections. This analysis highlights the robustness of the proposed scheme, focusing on its compression efficiency, decompression speed, and resource optimization in AR/VR applications. We provided evidence through comparative data and statistical modeling to support the strength of the scheme.

---

5. Figures 3 are very blurred. And the presentation of all figures and their explanation is somewhat ambiguous.

We replaced the blurred figures with high-resolution versions to improve clarity. Additionally, the captions and explanations for all figures have been revised and simplified. We avoided using statistical symbols such as (~) and opted for clear, text-based descriptions to enhance readability and precision. Each figure now clearly demonstrates the data it represents, improving the presentation and enhancing the overall understanding of the manuscript.

---

6. Add some comparative analysis with your method.

A detailed comparative analysis has been added to the results section, contrasting our proposed method with existing approaches. This section highlights the advantages and disadvantages of our method relative to others, showing where our approach offers significant performance improvements, especially in speed and memory efficiency.

Moreover, the entire paper is structured around a comprehensive comparison of various compression algorithms and development methods. This comparison is integral to the research, as it is the foundation for developing predictive mathematical models. These models are central to the paper's primary focus: accurately estimating performance characteristics such as memory usage, decompression speed, and overall application efficiency across different scenarios. The revised figures now contribute directly to this comparison, enhancing the reader's ability to grasp the study results and the algorithms' performance.

---

7. Clearly highlight the gaps and future work in the conclusion section.

We have expanded the conclusion to explicitly identify the current study's gaps and suggest future research directions. This includes investigating additional compression techniques, cloud-based testing environments, and real-time decompression methods, aligning with the journal's emphasis on comprehensive conclusions.

---

8. Add a summary of all results and show the pros and cons of it.

We have provided a clear summary of all results in the discussion section. This summary highlights the pros and cons of each compression method, weighing the trade-offs between compression efficiency, decompression speed, and resource usage. This comprehensive overview aids in understanding the strengths and limitations of each method.

---

9. The English of the paper should be polished further.

The manuscript has undergone a thorough English revision, improving grammar, sentence structure, and overall clarity. This has enhanced the readability and presentation of the paper, ensuring that the content is clearly communicated.

This revised manuscript, structured according to the PLOS ONE submission guidelines, addresses all the reviewer's comments and substantially improves the quality of the paper.
